# The Role of Lymph Node Fine-Needle Aspiration in Penile Cancer in the Sentinel Node Era

**DOI:** 10.1155/2011/383571

**Published:** 2011-03-30

**Authors:** Maria Carmen Mir, Olivia Herdiman, Damien M. Bolton, Nathan Lawrentschuk

**Affiliations:** ^1^Urology Unit, Department of Surgery, University of Melbourne, Austin Health, Australia; ^2^Ludwig Institute for Cancer Research, Austin Health, Melbourne VIC 3084, Australia

## Abstract

Penile squamous cell carcinoma (SCC) is an uncommon condition in Western countries. Inguinal lymph nodes dissection can be curative in 20%–60% of node positive patients. However, there is a high complication rates from the dissection, thus accurate diagnosis of inguinal lymph nodes metastasis is required. Current non invasive methods to detect lymph nodes metastasis are unreliable. Dynamic Sentinel Node Biopsy (DNSB), ultrasonography (US), and fine needle aspiration (FNA) cytology were proposed to in an attempt to detect sentinel lymph node (SLN). Despite the initial high rate of false negative results, recent DSNB showed improved survival compared to wait and see policy as well as reduced mortality compared to prophylactic inguinal lymphadenectomy. In addition, the US guided FNA shown 100% of specificity in detecting clinically occult lymph nodes metastasis. We proposed an algorithm for management of lymph nodes in penile cancer and suggest that FNA with US guidance should be performed in all high risk patients and that therapeutic dissection should be performed if findings are positive.

Penile squamous cell carcinoma (SCC) is an uncommon condition in western countries. It usually originates in the epithelium of the inner prepuce and glans. Penile SCC has an incidence of less than 1 per 100,000 males and accounting for 10%–20% of all malignancies in male patients in developing nations [1].

Of patients presenting with penile SCC, 30%–60% have enlarged lymph nodes in the groin. In about half of these patients, this is caused by metastatic invasion and in the other half by inflammatory reactions [2]. Nodal metastasis will develop in 10%–15% of the patients presenting with no clinical signs of nodal invasion [3].

Since SCC of the penis can be surgically cured despite the presence of inguinal lymph node metastasis, the appropriate management of the lymph node is extremely important in determining the treatment outcome. However, due to the relatively low incidence of penile SCC, limited number of patients' reports, and the rarity of prospective randomized trials, no clear guidelines for optimal treatment of patients with penile SCC and lymph nodes have been established.

Inguinal lymph node dissection can be curative in 20%–60% of node-positive patients. In node-negative men, inguinal lymph node dissection does not guarantee survival with a 5-year treatment failure rate of 5%–25% [4]. A report on 102 patients undergoing conservative management of the primary lesion (either brachytherapy or limited surgery) found that 32% of those with local relapse died compared with 75% of those with lymph node relapse, showing that nodal relapse remains the major cause of death [5]. 

Histologic grade and LVI are independent prognostic factors for occult metastasis in penile carcinoma. Although both predictors are incorporated into the current EAU guidelines, the stratification of patients needing a lymph node dissection is inaccurate. Graafland et al. describe that approximately 77% of high-risk patients in their study (188 of 245) would have had a negative bilateral inguinal lymphadenectomy. For the time being, DSNB is considered a more suitable staging method than EAU risk stratification for an accurate determination of patients who require lymph node dissection [6].

Additionally, inguinal lymph node dissection has a relatively high complication rate such as skin necrosis, seroma, lymphocoele, leg lymphedema, DVT, and femoral neurapraxia; hence, an accurate diagnosis of inguinal lymph node in the context of penile SCC treatment is required.

Currently, the noninvasive methods to detect lymph node metastasis are unreliable. However, there is a clinical need to find out real metastasis as soon as possible due to survival benefits demonstrated [4]. A surveillance policy in negative-node patients risks a noncurable disease once detected. On the contrary, an early inguinal lymphadenectomy in clinically node-negative patients is unnecessary in up to 80% [7].

Several methods of detecting a clinically occult metastasis have emerged: dynamic sentinel-node biopsy (DSNB), ultrasonography (US), and fine-needle aspiration (FNA) cytology of lymph nodes.

*DSNB* is performed by intradermal injection of technetium-99 m nanocolloid around the primary tumour, preoperative, and intraoperative identification of the sentinel lymph node (SLN) with the aid of intradermally administered patent blue dye and a gamma ray detection probe. The theoretical advantage of these techniques is that it is a functional rather than anatomical method of identifying sentinel lymph node (SLN). Anatomical studies showed that the SLN area has ≤7 lymph nodes located between the superficial epigastric and external pudendal veins [8]. Initially, sentinel node biopsy was not recommended because of a high rate of false-negative results (43%) [9]. However, recently, DSNB showed an improved survival versus a “wait and see” policy and reduced mortality compared to prophylactic inguinal lymphadenectomy [10]. Similarly, another European series demonstrated that DSNB has a 100% specificity and 95% sensitivity [11] and comparisons from two centres demonstrated that DSNB technique were reproducible [12]. Overall, DSNB is recommended for penile SCC with nonpalpable lymph nodes in EAU and SIU guidelines [13, 14]. Additionally, Graafland et al. [15] stated DSNB after resection of primary tumour as a suitable procedure to stage clinically node-negative penile carcinoma, with a 93% sentinel node visualization rate, identification of 100% and detection of occult metastasis in 12% of clinically node-negative groins.

*FNA Cytology* of inguinal lymph nodes has previously been evaluated under different clinical circumstances as a mean of determining metastatic involvement from penile carcinoma. Kumar et al. [16] reported 100% sensitivity of FNA for the detection of metastatic penile carcinoma in palpable inguinal lymphadenopathy in a series of 28 patients. The FNA cytology was done at the time of inguinal node dissection and not before antibiotic therapy. Despite the sensitivity, they suggested that patients with negative FNA should be subjected to medial inguinal lymph node biopsy to overcome the risk of a false negative cytologic assessment.

Accurate sampling by FNA is often cited as an issue when many nodes are enlarged, as is often the case [16]. This has lead to other methods of accurately identifying nodes for cytologic assessment using lymphangiography and CT scanning, which have been largely been discouraging [17–19]. Just recently, Graafland et al. used CT scan imaging to describe the inguinal node features for positivity of involvement (central nodal necrosis and/or an irregular nodal border) [20].

Recently, Saisorn et al. [21] demonstrated that FNA of palpable inguinal lymphadenopathy (no ultrasound was performed during FNA) under local anaesthetic at the time of biopsy of the primary penile SCC is safe and accurate in predicting metastatic disease. They assessed a total of 16 patients with penile cancer and palpable lymph nodes, performing at the time of primary lesion biopsy, an FNA of palpable lymph nodes, partial penectomy, and synchronous bilateral inguinal lymph node dissection. By using the algorithm described by these authors, 52% of the patients could have spared the six-week period of antibiotic treatment before proceeding to inguinal lymph node dissection, hence, reporting a better survival on the long-term followup.

In relation to nonpalpable inguinal lymph nodes, Kroon et al. [22] added the US for an accurate FNA. These authors performed 34 groins USs and FNAs previous to DSNB. The sensitivity of US-guided FNA in detecting clinically occult lymph node metastases was 39% with 100% specificity. This number showed to be similar to the ones used in other cancer pathologies (breast, melanoma), where US pre- DSNB is clearly established. This concludes that US-FNA cannot replace DNSB but is a useful tool for preoperative screening of clinically node-negative groins in patients scheduled to undergo DNSB. Up to 10% of groin DNSB can be avoided in favour of direct inguinal lymph node dissection. In the effort of improving the efficiency of US-FNA, these authors suggested using an echogenic contrast to differentiate pathologic features on lymph nodes as well as increasing the US probe frequency.

Moreover, US-FNA has a role during followup after DNSB as earlier detection of recurrences might be expected. However, no randomized control trials have proven that statement.

In conclusion, we suggest/recommend that FNA with US guidance may be performed in all patients with or without clinically palpable nodes especially in those who are at high risk of lymph nodes involvement (Figure 1). If the findings are positive, therapeutic, rather than diagnostic, inguinal lymph node dissection can be performed [14].

## Figures and Tables

**Figure 1:**
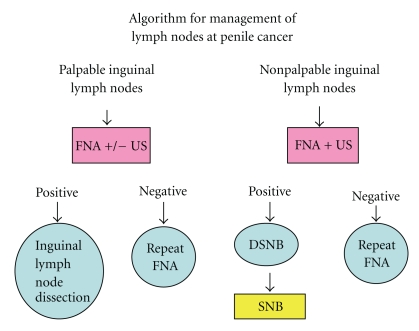


## References

[1] Parkin DM, Ferlay J, Curado M-P (2010). Fifty years of cancer incidence: CI5 I-IX. *International Journal of Cancer*.

[2] Abi-Aad AS, DeKernion JB (1992). Controversies in ilioinguinal lymphadenectomy for cancer of the penis. *Urologic Clinics of North America*.

[3] Horenblas S (2001). Lymphadenectomy for squamous cell carcinoma of the penis. Part 1: diagnosis of lymph node metastasis. *BJU International*.

[4] Pandey D, Mahajan V, Kannan RR (2006). Prognostic factors in node-positive carcinoma of the penis. *Journal of Surgical Oncology*.

[5] Soria JC, Fizazi K, Piron D (1997). Squamous cell carcinoma of the penis: multivariate analysis of prognostic factors and natural history in a monocentric study with a conservative policy. *Annals of Oncology*.

[6] Graafland NM, Lam W, Leijte JAP (2010). Prognostic factors for occult inguinal lymph node involvement in penile carcinoma and assessment of the high-risk EAU subgroup: a two-institution analysis of 342 clinically node-negative patients. *European Urology*.

[7] Bevan-Thomas R, Slaton JW, Pettaway CA (2002). Contemporary morbidity from lymphadenectomy for penile squamous cell carcinoma: the M.D. Anderson cancer center experience. *Journal of Urology*.

[8] Kroon BK, Horenblas S, Nieweg OE (2005). Contemporary management of penile squamous cell carcinoma. *Journal of Surgical Oncology*.

[9] Pettaway CA, Pisters LL, Dinney CPN (1995). Sentinel lymph node dissection for penile carcinoma: the M.D. Anderson Cancer Center experience. *Journal of Urology*.

[10] Lont AP, Horenblas S, Tanis PJ, Gallee MPW, Van Tinteren H, Nieweg OE (2003). Management of clinically node negative penile carcinoma: Improved survival after the introduction of dynamic sentinel node biopsy. *Journal of Urology*.

[11] Leijte JAP, Kroon BK, Valdés Olmos RA, Nieweg OE, Horenblas S (2007). Reliability and safety of current dynamic sentinel node biopsy for penile carcinoma. *European Urology*.

[12] Leijte JAP, Hughes B, Graafland NM (2009). Two-center evaluation of dynamic sentinel node biopsy for squamous cell carcinoma of the penis. *Journal of Clinical Oncology*.

[13] Algaba F, Horenblas S, Pizzocaro-Luigi Piva G, Solsona E, Windahl T (2002). EAU guidelines on penile cancer. *European Urology*.

[14] Heyns CF, Fleshner N, Sangar V, Schlenker B, Yuvaraja TB, Van Poppel H (2010). Management of the lymph nodes in penile cancer. *Urology*.

[15] Graafland NM, Valdés Olmos RA, Meinhardt W (2010). Nodal staging in penile carcinoma by dynamic sentinel node biopsy after previous therapeutic primary tumour resection. *European Urology*.

[16] Kumar MPS, Ananthakrishnan N, Prema V (1998). Predicting regional lymph node metastasis in carcinoma of the penis: a comparison between fine-needle aspiration cytology, sentinel lymph node biopsy and medial inguinal lymph node biopsy. *British Journal of Urology*.

[17] Scappini P, Piscioli F, Pusiol T (1986). Penile cancer: aspiration biopsy cytology for staging. *Cancer*.

[18] Wajsman Z, Gamarra M, Park JJ (1982). Transabdominal fine needle aspiration of retroperitoneal lymph nodes in staging of genitourinary tract cancer (correlation with lymphography and lymph node dissection findings). *Journal of Urology*.

[19] Luciani L, Piscioli F, Scappini P, Pusiol T (1984). Value and role of percutaneous regional node aspiration cytology in the management of penile carcinoma. *European Urology*.

[20] Graafland NM, Teertstra HJ, Besnard APE, van Boven HH, Horenblas S (2011). Identification of high risk pathological node positive penile carcinoma: value of preoperative computerized tomography imaging. *Journal of Urology*.

[21] Saisorn I, Lawrentschuk N, Leewansangtong S, Bolton DM (2006). Fine-needle aspiration cytology predicts inguinal lymph node metastasis without antibiotic pretreatment in penile carcinoma. *BJU International*.

[22] Kroon BK, Horenblas S, Deurloo EE, Nieweg OE, Teertstra HJ (2005). Ultrasonography-guided fine-needle aspiration cytology before sentinel node biopsy in patients with penile carcinoma. *BJU International*.

